# Comparative physiological and transcriptomic analysis of pear leaves under distinct training systems

**DOI:** 10.1038/s41598-020-75794-z

**Published:** 2020-11-03

**Authors:** Zheng Liu, Liyuan An, Shihua Lin, Tao Wu, Xianming Li, Junfan Tu, Fuchen Yang, Hongyan Zhu, Li Yang, Yinsheng Cheng, Zhongqi Qin

**Affiliations:** 1 Research Institute of Fruit and Tea, Hubei Academy of Agricultural Sciences, Wuhan, 430064 China; 2 College of Life Sciences, Wuhan University, Wuhan, 430072 China

**Keywords:** Molecular biology, Physiology, Plant sciences

## Abstract

Canopy architecture is critical in determining the light interception and distribution, and subsequently the photosynthetic efficiency and productivity. However, the physiological responses and molecular mechanisms by which pear canopy architectural traits impact on photosynthesis remain poorly understood. Here, physiological investigations coupled with comparative transcriptomic analyses were performed in pear leaves under distinct training systems. Compared with traditional freestanding system, flat-type trellis system (DP) showed higher net photosynthetic rate (*P*_N_) levels at the most time points throughout the entire monitored period, especially for the interior of the canopy in sunny side. Gene ontology analysis revealed that photosynthesis, carbohydrate derivative catabolic process and fatty acid metabolic process were over-represented in leaves of DP system with open-canopy characteristics. Weighted gene co-expression network analysis uncovered a significant network module positive correlated with *P*_N_ value. The hub genes (*PpFKF1* and *PpPRR5*) of the module were enriched in circadian rhythm pathway, suggesting a functional role for circadian clock genes in mediating photosynthetic performance under distinct training systems. These results draw a link between pear photosynthetic response and specific canopy architectural traits, and highlight light harvesting and circadian clock network as potential targets for the input signals from the fluctuating light availability under distinct training systems.

## Introduction

Light is a primary energy source to accumulate dry matter in plant and also an important ambient signal for growth and development^1,2^. Both light quality and light intensity have been shown to have a diverse range of effects on plant physiology and biochemisty^3,4^. It is therefore not surprising that plants have evolved several strategies to optimize the photosynthetic machinery to light changes^5,6^. In the field of fruit tree cultivation and management, training systems generally determine tree architecture, which represent spatial strategy for light interception and distribution within the canopy, and therefore influence photosynthetic efficiency and productivity^7–9^. The net photosynthetic rate (*P*_N_) is an important parameter that can be used to analyze canopy receptive competence of photosynthetically active radiation (PAR), and help selection what is the optimal canopy architecture through pruning and thinning^10^. However, the effect of architectural heterogeneity on photosynthesis has received limited attention.

Training systems exhibit significant spatio-temporal variability due to architectural traits such as canopy position, crop load and canopy sides (sunny/shady side), and therefore lead to photosynthetic variability. A good training system can optimize canopy structure, which could improve microclimate conditions and affect overall canopy photosynthetic productivity^9,11^. In most natural canopies, the light availability decreases from the exterior to the interior of the canopy^12^. Light environment is an important determinant of nitrogen per unit leaf area and the leaf nitrogen partitioning within the canopy^13^. The heterogeneous light environment and nitrogen distribution could cause different canopy photosynthetic responsiveness^14^. Current evidences have indicated that the sink effect on photosynthesis may operate through feed back/ feed forward regulatory controls, mostly depending upon the carbohydrate levels in leaves^15,16^. Fruit set and retention generally stimulates photosynthetic activity, whereas removal of the sink demand was found to have a contrast effect on source leaf photosynthesis in fruit species^17,18^. During daylight, distinctly different light intensities were showed between opposite canopy sides. East is a sunny side during the morning and a shady one during the afternoon; the opposite occurs on the West side. Shading may change the light spectrum, and decrease shoots photosynthesis^19^. Shady leaves must catch photons as efficiently as possible, whereas the sun leaves may protect themselves against the high light and temperature that may damage their photosynthetic structure^20,21^. Therefore, it is useful to understand how such architectural traits affect photosynthesis in order to fully to comprehend this complicated physiological process, which will be helpful for improving fruit quality through the correct choice of cultural practices.

Traditional freestanding systems, such as spindle and delayed-open central leader, are widely employed in economic deciduous fruit tree pear. However, the high labor requirements of traditional training systems are driving innovation in orchard systems. Hence, flat-type trellis systems, such as ‘Joint Tree’ and ‘Double Primary Branches Along the Row’, have the advantage of reduced labor and thus been developed for pear cultivation^9,22^. These arrangements have also been found to allow trees to receive sunlight uniformly and achieve relatively uniform fruit quality. Better understanding of the effect of training systems on photosynthesis at the physiological and molecular level could provide basic information and important guidance for orchard management, and thus realize the promised increased in profitability in orchard.

Fluctuating light is ubiquitous within canopy, and all leaves are shaded to some degree during their lifecycle. Shaded light is characterized by low light intensity (decreased PAR), a low red/far-red light ratio and low blue light^23–25^. PAR is a primary driving force for photosynthesis, and different wavelengths of light are considered to be important environmental signals^25–27^. Circadian clock is an internal timing mechanism that allows plants as sessile organisms to synchronize with environmental cues^28^. Phytochromes and cryptochromes are most well-characterized circadian clock-mediating photoreceptors, sensing and responding to changes in red, far-red and blue wavelengths of light^28^. With the rapid development of molecular biotechnologies and bioinformatics, some genes associated with responding to light variability have been identified. For example, phytochrome was found to interact with a bHLH transcription factor *PIF3* that have been established as transcriptional regulators of *LHY* and *CCA1*, directly linking the light signals and circadian clock regulatory networks^29,30^. A dynamic proteomics analysis of maize leaves revealed the diurnal light regulation of diverse biological processes including photosynthesis, carbon fixation and the TCA cycle^31^. A significantly decreased expression of genes that were related to starch and sucrose biosynthesis, glycolysis, TCA cycle and mitochondrial electron transport was observed in cassava leaves under natural shady condition^19^. Although these previous studies are highly valuable for surveying key regulatory genes and biological processes responding to light variability, it is difficult to apply these reported results to infer that occur in other complex agricultural conditions.

In this study, to investigate the potential mechanism affect pear photosynthesis responses with distinct training systems, we conducted physiological and transcriptomic surveys to capture progressive stages of photosynthetic differentiation between traditional freestanding system (delayed-open central leader, SP) and flat-type trellis system (Double Primary Branches Along the Row, DP). Weighted gene co-expression network analysis was constructed to identify hub gene associated with photosynthetic performance of this perennial tree under distinct training systems. We also investigated the effects of training systems on photosynthetic pigments, photosynthetic nitrogen-use efficiency (PNUE), soluble protein and photosynthetic enzyme activity. These analyses revealed that heterogeneous environment within canopy causes dramatic differences in the photosynthetic physiology and transcriptional levels among various architectural traits leaves. We identified the biological process and pathways enriched in distinct training systems and key candidate genes that apparently regulate photosynthetic level. This integrative view may contribute to our understanding of the pear photosynthetic responses to changes in tree architectures, and provide a valuable reference for pear cultivation and improvement efforts.

## Materials and methods

### Plant materials

Ten-year-old ‘Wonhwang’ (*Pyrus pyrifolia* Nakai cv. Wonhwang) pear trees growing in the experimental orchard (30.292°N, 114.143°E) of Hubei Academy of Agricultural Sciences, Research Institute of Fruit and Tea, were used for this study. The planting distance was 3 m between the rows and 4 m between the trees. The trees in this experimental orchard received fertilizers, irrigation, and chemical thinners in accordance with the local recommendations. Trees have been trained to SP or DP system as described previously^9^. Briefly, the SP system consists of one central stem and 3–5 primary branches that are upright and located at about 0.5–2 m from the ground. Both the central stem and primary branches have several smaller sub-branches. The DP trellis system is near to a Y-shaped system, which features a support structure consisting of one central stem and two primary branches bent in opposite directions along the row. The height of the trellis is 1.7–1.8 m. At planting, trees are headed at 1.2–1.3 m above the ground so that they would have an upright central leader at the base. Heading back produced many strong shoots that were selected and trained to the two proper arms of the trellis system. Each primary branch with an incline of 45° above the horizontal has equally spaced sub-branches that were naturally tied on horizontal steel wires suspended from concrete posts. The experiment was a randomized complete block design with three replications. Trees within each block were randomly selected, which represented biological replicates per training system. Each tested tree was divided into eight leaf locations, i.e., sunny side (SU)-interior part of the canopy (IN)-vegetative shoots (VE), SU-IN-fruiting shoots (FR), SU-exterior part of the canopy (EX)-VE, SU-EX-FR, shady side (SH)-IN-VE, SH-IN-FR, SH-EX-EV, SH-EX-FR. IN and EX were approximately 0–1.0 m and more than 1.0 m away from the trunk, respectively.

### Photosynthetic measurements

Diurnal courses of photosynthetic parameters including *P*_N_ and leaf temperature (T) were measured using the portable TPS-2 photosynthesis system (PP. Systems Inc., USA). All measurements were carried out at regular intervals of 2 h (n = 5), between 08:00 and 16:00 on sunny and clear days, during the spring–summer productive period including 15 DAF (day after flowering), 45 DAF, 75 DAF and 105 DAF. Leaves on the East side at 8:00, 10:00, 12:00 and leaves on the West side at 14:00, 16:00 are considered as sun leaves, while leaves on the West side at 8:00, 10:00, 12:00 and leaves on the East side at 14:00, 16:00 are considered as shady leaves. Photosynthetic photon flux density (PPFD) was measured with LI-180 Spectrometer (LI-COR Inc., USA) at 5 cm above the surface of the leaves in specific location. All PPFD measurements were taken every 2 h between 08:00 and 16:00 on sunny and clear day (105 DAF). For each biological replicate of each leaf location, photosynthetic parameters were measured on three leaves (three technical replicates), which were youngest, fully expanded sixth leaves from 1-year-old shoots, at five time points of the day. Statistical analyses were performed with a model of one-way ANOVA analyses (IBM SPSS Statistics 19 software), followed by the Duncan’s multiple range tests at *p* < 0.01 as described previously^32^.

### RNA isolation and RNA-seq

Leaf samples from each leaf location were collected at three important growth stages, i.e., 45 DAF, 75 DAF and 105 DAF, and immediately frozen in liquid nitrogen and stored at − 80 °C until use. For each sample, three biological replicates were performed. Total RNA was extracted from the frozen leaves using RNAprep Pure Plant Kit (Polysaccharides & Polyphenolics-rich) (Tiangen, China) according to the manufacturer’s protocol, followed by integrity evaluation using Bioanalyzer 2100 (Agilent, Germany). For each development stage of each training system, the RNAs from SU-IN-VE and SU-IN-FR at 10:00 were uniformly pooled and used as a single sample for the transcriptome sequencing.

mRNA-seq libraries were constructed and sequenced on an Illumina HiSeq 2500 platform by Personal Biotechnology Co., Ltd. (Shanghai, China). Raw reads were processed by stripping the adaptor sequences and ambiguous nucleotides using SeqPrep (https://github.com/jstjohn/SeqPrep) and Sickle (https://github.com/najoshi/sickle). Reads with quality score less than 20 and lengths below 30 bp were removed. The clean reads were aligned to the reference genome sequence of pear (*Pyrus bretschneideri* Rehd.) via HISAT2 (https://ccb.jhu.edu/software/hisat2/index.shtml) with default settings^33^. The sequence data were deposited at NCBI Sequence Read Archive under the accession number PRJNA579772. Gene expression levels were calculated according to the FPKM (Fragments Per Kilobases per Millionreads) method for genes in each library^34^. Correlation analysis for gene expression levels between any two samples was performed by spearman algorithm. Differentially expressed genes (DEGs) were detected using DESeq2^35^, and Benjamini-Hochberg’s method was used to control the false discovery rate (FDR). Only genes with absolute value of log_2_(fold change) ≥ 1 and adjusted *p* value < 0.05 were considered significantly differentially expressed. We analyzed the gene relationships and identified the overlapping DEGs using VennDiagram^36^.

### Quantitative real time-PCR (qRT-PCR) analysis

To validate the RNA-seq results, expression of selected genes was determined by qRT-PCR analysis. One microgram of total RNA was reverse-transcribed using RevertAid First Strand cDNA Synthesis Kit (Fermentas, USA). qRT-PCR primer pairs specific to selected genes were designed by Primer Premier 5.0 software (Supplementary Table S1). The primers were further confirmed with a melting curve analysis after amplification of each tested genes. Two reference genes, i.e. *PpSKD1* and *PpYLS8*, which proved to be stably expressed in leaves during different growth stages under distinct pear training systems, were used as suitable internal controls to normalize the qRT-PCR data^9^. Relative quantification was calculated based on the Ct method (2^−△△*C*T^). Three independent biological replicates for each sample and three technical replicates of each biological replicate were performed.

### Functional annotation, pathways and transcription factors (TFs) analysis

In order to identify the significantly enriched Gene Ontology (GO) terms, each of the DEG lists were mapped into GO terms in the database (https://www.geneontology.org/). Go functional enrichment analysis was performed using Goatools software by Fisher’s exact test^37^. Terms are considered enriched at *p* < 0.001. Kyoto Encyclopedia of Genes and Genomes (KEGG) pathways enriched analysis were performed (https://www.genome.jp/kegg/), using the criterion of a Benjamini–Hochberg corrected *p* value < 0.05^38–40^. Up-regulated and down-regulated TFs were identified and classified into different families using PlantTFDB v4.0 (https://planttfdb.cbi.pku.edu.cn), with a threshold E-value < 1E−5.

### Weighted gene co-expression network analysis (WGCNA)

A network analysis based on gene-to-trait correlations was performed using WGCNA R package^41^. As it is believed that low expressed and non-changing genes provide limited information in a co-expression network building, 13,521 pear genes were selected based on their expression values ≥ 1 FPKM in one or more samples and coefficient of variation (CV) ≥ 0.1. A soft threshold value, power of 6, was used to transform the adjacency matrix to meet the scale-free topology criteria for optimal clustering. Modules whose eigengenes were highly correlated were merged with a mergeCutHeight of 0.25. The minimal module size was set to 30 genes. Module-trait associations were assessed by calculating the spearman’s correlations between the module eigengenes (MEs) and the *P*_N_ values. Top 30 genes in intramodular connectivity ranking were counted as hub genes in a given module. The network for hub genes was visualized using the Highcharts (v6.0.4) (https://www.highcharts.com/).

### Measurement of photosynthetic pigments, PNUE, soluble protein and Rubisco activase (PpRCA) enzyme activity

Photosynthetic pigments were extracted from 0.1 g leaf sample with 1 ml acetone/ethanol mixture (2:1, v/v). The extraction was performed in dark until the samples were completely white. Concentrations of chlorophyII (Chl) *a*, Chl *b* and carotenoid (Car) were determined by absorbance values, at respective wavelengths of 663, 645 and 470 nm.

After completing the *P*_N_ measurements, the same leaves were excised to measure leaf mass per area. The leaves were then oven-dried at 60 °C for one week to determine their dry mass, and the samples were digested with H_2_SO_4_–H_2_O_2_ at 260–270 °C. The leaf nitrogen content was measured with a K1160 Automatic Kjeldahl analyzer (Hanon, China). PNUE was defined as *P*_N_ divided by leaf nitrogen content per unit area^42^.

Soluble protein content of leave was measured by the Coomassie brilliant blue G250 method. Leaf sample was homogenized in 1 ml distilled water, and the solution was centrifuged at the speed of 10,000 rpm for 10 min at 4 °C. The supernatant was used for colorimetric assay of soluble protein content at a wavelength of 595 nm. Standard curve were prepared with bovine serum albumin (Sigma, ultra 99%).

PpRCA enzyme activity was determined by an enzyme-linked immunosorbent assay (ELISA) using Rubisco Activase Assay Kit according to the manufacturer instructions. The absorbance was measured at 450 nm. The activity of RCA enzyme in samples was calculated by the standard curve.

All components described above were measured between DP and SP system. SU-IN leaf samples were collected from each replicate (n = 8) at 10:00 of 105 DAF. A paired two-tailed Student’s t-test (IBM SPSS Statistics 19 software) was performed to compare between the distinct training systems. Statistical significance was defined as *p* ≤ 0.001 (★★★), 0.001 < *p* ≤ 0.01 (★★), 0.01 < *p* ≤ 0.05 (★).

## Results

### Spatial and temporal variability of biochemical photosynthetic parameters under distinct training systems

**Figure 1:**
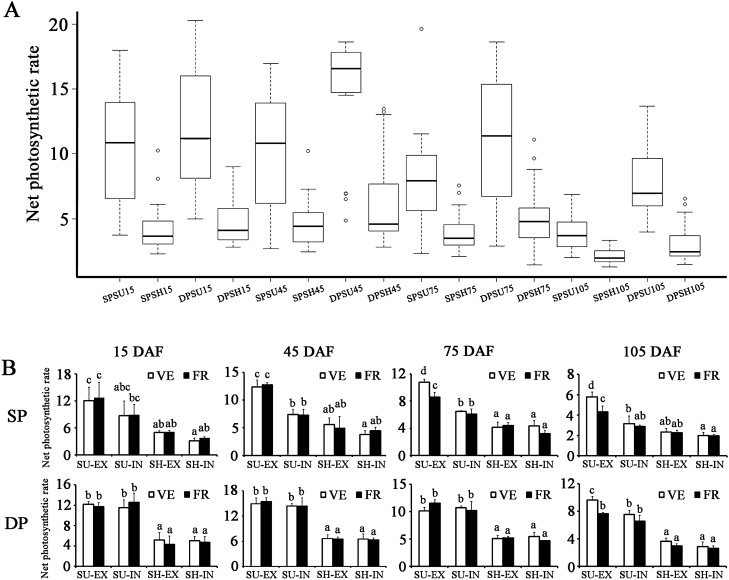
Changes in net photosynthetic rate (*P*_N_) in pear leaves at four growth stages. (**A**) Box plots of the *P*_N_ values from 16 leaf samples. Each sample contained the leaf tissues from the four typical canopy locations, i.e. interior part of the canopy (IN)-vegetative shoots (VE), IN-fruiting shoots (FR), exterior part of the canopy (EX)-VE, EX-FR, at five time points. SPSU15/45/75/105: sun leaves of SP system (traditional freestanding system) at 15/45/75/105 DAF (day after flowering), SPSH15/45/75/105: shade leaves of SP system at 15/45/75/105 DAF, DPSU15/45/75/105: sun leaves of DP system (flat-type trellis system) at 15/45/75/105 DAF, DPSH15/45/75/105: shade leaves of DP system at 15/45/75/105 DAF. The box represents the 25th and 75th percentiles of the data. A *line across the box* is depicted as the median. *Whiskers* go from the minimal to maximal value. *Circles* indicate outliers. (**B**) *P*_N_ values of various types of leaves. SU-EX: sun leaves in EX, SU-IN: sun leaves in IN, SH-EX: shade leaves in EX, SH-IN: shade leaves in IN. Bars represent the standard errors. The capital letters above the bars indicted significant differences (*p* < 0.01).

To understand the response of photosynthesis to variable canopy positions, the *P*_N_ fluctuations of eight typical canopy locations were monitored from 15 to 105 DAF. The leaf *P*_N_ varied between 1.30 and 20.27 µmol m^2^ s^−1^ during the time period (Fig. 1A). Among these, the sun leaves of DP at 45 DAF (DPSU45) showed the highest daily median value (16.57 µmol m^2^ s^−1^), whereas the shade leaves of SP at 105 DAF (SPSH105) presented the lowest average *P*_N_ (1.97 µmol m^2^ s^−1^). The difference may due to the sensitivity to light depending on the leaf growth stages and spatio-temporal variations in light exposure.

The leaf *P*_N_ in sunny side displayed relatively higher variation, with *P*_N_ values 2.00–20.27 µmol m^2^ s^−1^ (Fig. 1A). In contrast, the leaf *P*_N_ in shady side showed relatively narrow range spanning 1.30–13.47 µmol m^2^ s^−1^. The rates of *P*_N_ were markedly higher in sun leaves than in shade leaves (Fig. 1B), which is probably due to higher light intensity in sunny side.

Fruiting leaves and vegetative counterparts had similar photosynthetic capacity at the most time points throughout the entire monitored period, with a few minor exceptions that *P*_N_ value of SU-EX exhibited higher levels in vegetative leaves than that in fruiting leaves at 75 DAF (SP) and 105 DAF (Fig. 1B).

**Figure 2:**
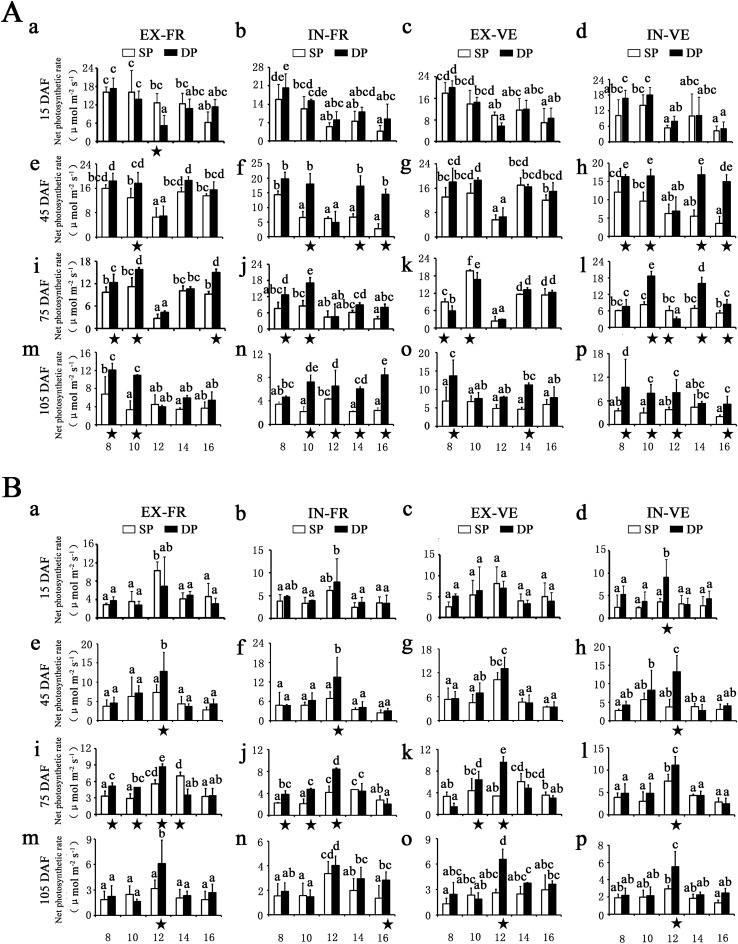
Diurnal variation in net photosynthetic rate (*P*_N_) of eight typical canopy locations at five time points. The diurnal patterns of the *P*_N_ in sunny side (**A**) and shady side (**B**) were displayed, respectively. EX-FR: fruiting leaves in the exterior part of the canopy, IN-FR: fruiting leaves in the interior part of the canopy, EX-VE: vegetative leaves in the exterior part of the canopy, IN-VE: vegetative leaves in the interior part of the canopy, SP: traditional freestanding system, DP: flat-type trellis system, DAF: day after flowering. Each value represents mean ± standard deviation (n = 9). The capital letters above the bars indicted significant differences (*p* < 0.01). Asterisks below indicate significantly higher levels of *P*_N_ between SP and DP system.

To analysis the diurnal profiles of photosynthetic activity, the leaf temperature, PPFD and *P*_N_ parameters were monitored along with five time points throughout daylight (Fig. 2 and Supplementary Figure S1). Similar changing patterns and levels of leaf temperature were observed between sunny side and shady side (Supplementary Figure S1A). Leaf temperature was increased rapidly in the morning and showed a relatively high peak value at 12:00 h or 14:00 h, and then slight decreased thereafter. The diurnal patterns of the PPFD also showed a variation similar to the changes of leaf temperature, which exhibited its daily maximum at solar noon, except for SP leaves in the interior part of the canopy, where PPFD values remained relatively constant during the course of the day (Supplementary Figure S1B). When looking at the discrimination between the distinct training systems, significantly higher PPFD values were observed in DP leaves than in SP leaves under SU/SH-IN and SH-EX conditions. Under the fluctuating light intensity and temperature, the diurnal patterns of *P*_N_ showed a strong midday depression in most sun leaves (Fig. 2A). In shady side, the diurnal course of *P*_N_ showed approximately ‘dome shaped’ pattern (Fig. 2B). Most of them reached a maximum at noon, whereas others displayed no significant variation trend.

For DP system, leaf *P*_N_ was not statistically different between exterior and interior part of trees at the most canopy positions (Fig. 1B). In contrast, in sunny side, the SP system displayed significantly higher values in exterior part of the canopy except at 15 DAF stage. More importantly, *P*_N_ showed relatively higher levels in DP compared with SP, especially for the interior part of the canopy (Figs. 1A and 2), indicating that mutual shading may be more severe in SP system. The difference in *P*_N_ between SP and DP training systems appeared at the 12:00 h of 15 DAF but became more evident at the later stages (45 DAF, 75 DAF and 105 DAF) (Fig. 2A). It is conceivable that the DEGs between SP and DP leaves in SU-IN at the last three stages may play important roles in determining the differences of photosynthetic efficiency under distinct training systems; hence, these were subject to detailed investigation.

### Overview of the RNA-Seq data

**Figure 3:**
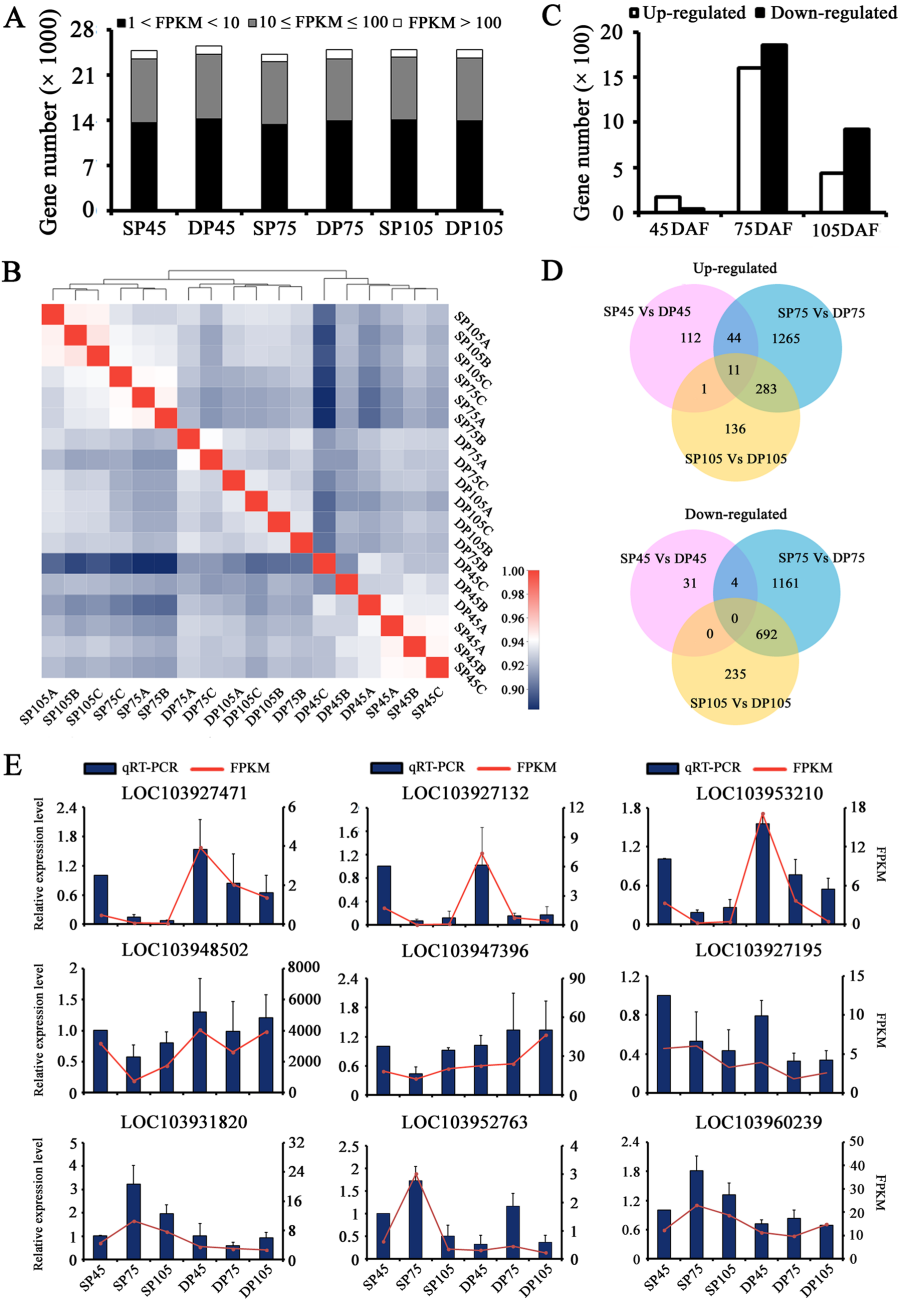
Global view of the RNA-seq expression data at three growth stages in pear. (**A**) Overview of gene expression in different samples. Samples from the SP (traditional freestanding system) and DP (flat-type trellis system) were collected at 45 DAF (day after flowering), 75 DAF and 105 DAF. FPKM: Fragments Per Kilobases per Millionreads. The white, grey and dark segments in each stacked bar indicate the distribution of different expression levels. (**B**) Hierarchical clustering analysis of all expressed genes across the different samples. Each column and row corresponds to one sample. Color gradient from red-to-blue indicates similarity degree change from high to low. (**C**) The total number of up- and down-regulated genes at three growth stages. (**D**) Venn diagram showing the number of genes with differentially expressed levels between SP and DP systems at the three growth stages. (**E**) Verification of RNA-seq results by qRT-PCR. Error bars indicate the standard deviation from three biological and technical replicates of qRT-PCR analysis. The Y-axises show the relative gene expression level analyzed by qRT-PCR (blue bars, left) and corresponding expression data of RNA-seq (red line, right).

To explore how light availability affected by distinct canopy structures impacts transcription at the genomic scale, we performed RNA-seq analysis comparing gene expression changes between DP and SP leaves in SU-IN at three successive stages (45 DAF, 75 DAF and 105 DAF) of leaf development. After removing low quality reads, a total of 41.85–53.26 million clean reads were generated from each library (Supplementary Table S2). The percentages of Q20 were higher than 98.3% in all libraries, representing high quality sequencing. We found 74.49–78.38% of the clean reads in the libraries were mapped onto the pear reference genome. Based on FPKM value, we determined the number of genes expressed (FPKM > 1) in individual leaf sample (Fig. 3A). In total, 24,734, 24,203 and 24,886 genes were found to be expressed in SP leaves at 45 DAF, 75 DAF and 105 DAF, respectively. Similarly, 25,524, 24,891 and 24,937 genes were identified in the samples from the respective stages of DP leaves. We also observed similar distributions of gene expression levels across all samples. Approximately 55.7% of expressed genes were in the 1–10 FPKM range, and 39.2% of expressed genes were in the range 10–100 FPKM.

To investigate the relationships among the samples used in this study, we performed hierarchical clustering based on the whole-gene expression data set. The result showed that all samples had a high degree of similarity (more than 0.883) and clustered in three discrete groups (Fig. 3B). This analysis clearly revealed the distinctness of the early stage (45 DAF) tissues from the later stages (75 DAF and 105 DAF) tissues, indicating the presence of different transcriptional programs. In addition, among the two later stages samples, the SP system leaf samples (SP75A/B/C and SP105A/B/C) clustered closely. Likewise, DP system leaf samples (DP75A/B/C and DP105A/B/C) also showed very tight clustering. This indicates distinct transcriptional programs active between the SP and DP samples, but somewhat similar within each other.

All the stages of leaf development analyzed in this transcriptome study showed significantly different photosynthetic rates, which imply dramatic differences in their transcriptional programs. Three pairwise transcriptome comparisons were performed between the SP and DP leaves. A total of 203 (168 up-regulated and 35 down-regulated), 3460 (1603 up-regulated and 1857 down-regulated) and 1358 (431 up-regulated and 927 down-regulated) genes were significantly differentially expressed at 45 DAF, 75 DAF and 105 DAF, respectively (Fig. 3C). Consistent with correlation analysis results, striking differential expression was observed at the two later stages, which may be due to distinct canopy architectures present significantly different microclimate during that growth stages.

Venn diagram analysis showed that only 11 genes were commonly up-regulated in the three stages (Fig. 3D). There was no down-regulated gene overlapped among all the three stages, but 4 and 692 genes were commonly down-regulated in 45 DAF/75 DAF pair and 75 DAF/105 DAF pair, respectively. More overlapped genes were observed between 75 and 105 DAF than between 45 and 75 DAF in both up-regulated and down-regulated genes.

The expression levels of nine interesting DEGs (five up-regulated and four down-regulated) were validated by qRT-PCR (Fig. 3E), all of which are known to be related to electron transport (*PpPPO*, polyphenol oxidase, LOC103927471), carbohydrate metabolic process (*PpG3PDH*, glycerol-3-phosphate dehydrogenase, LOC103927132 and LOC103953210), photorespiration (*PpRbcS*, the small subunit of ribulose-1,5-bisphosphate carboxylase, LOC103948502), as well as some TFs (*PpPIF4*, putative bHLH transcription factor, LOC103947396; *PpDOF*, putative DNA-binding one zinc finger transcription factor, LOC103927195; *PpMYB4*, putative MYB transcription factor, LOC103931820; *PpERF*, putative ethylene responsive factor, LOC103952763; *PpHSF*, putative heat stress transcription factor, LOC103960239). qRT-PCR expression profiles of the selected genes were consistent with the deep sequencing results, indicating that the RNA-seq data in this study is reliable.

### Analysis of differentially expressed genes (DEGs) responding to microclimate conditions under distinct training systems.

**Figure 4:**
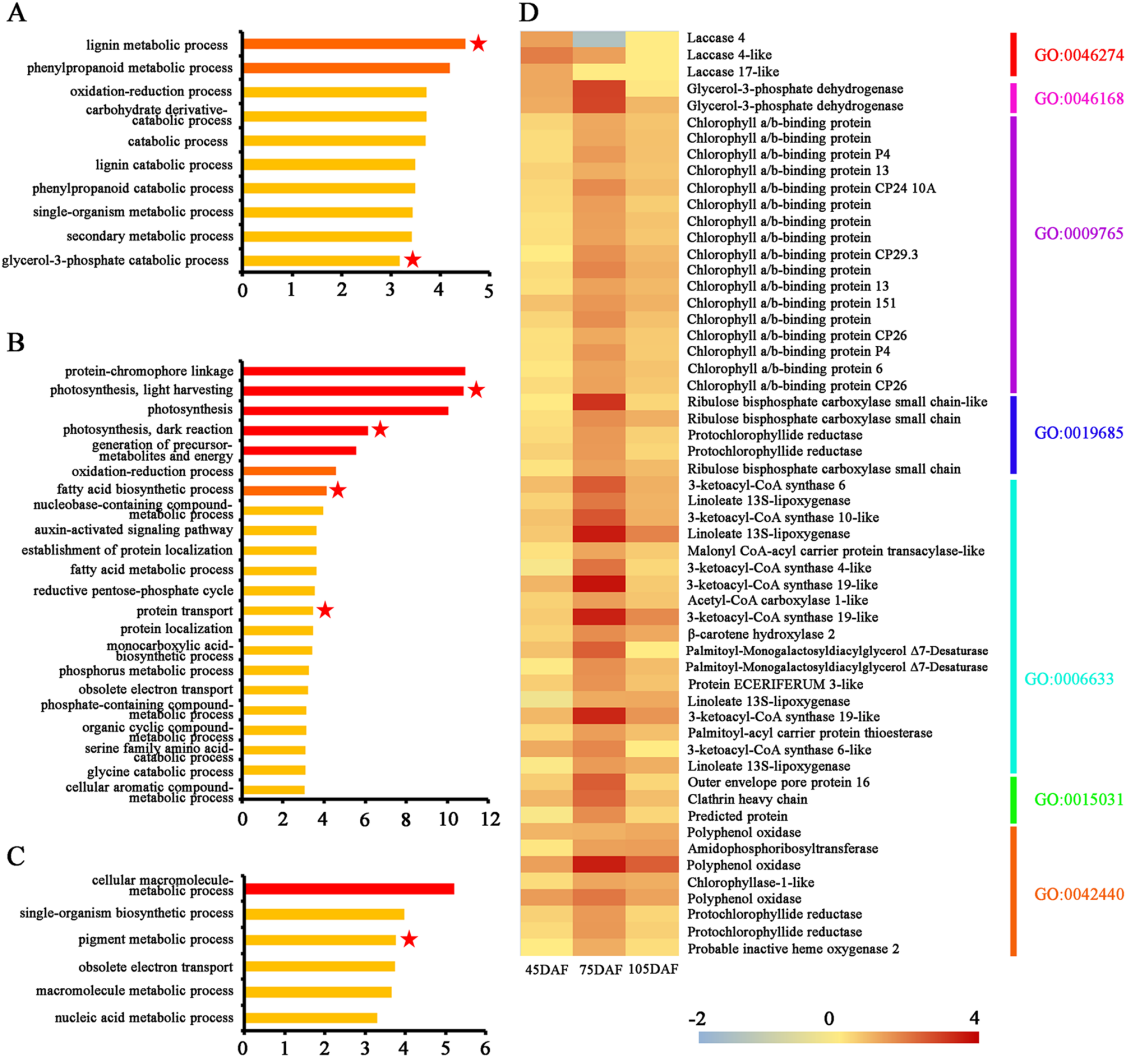
Enriched Gene Ontology (GO) biological process based on up-regulated genes at the three growth stages. Go enrichment analysis was performed based on the up-regulated genes between SP (traditional freestanding system) and DP (flat-type trellis system) at 45 DAF (**A**), 75 DAF (**B**) and 105 DAF (**C**), with *p* value < 0.001 as significant. DAF: day after flowering. The x-axis is the – log_10_ (*p* value). Color gradient from red-to-yellow indicates decreasing significance levels, i.e. red = most, orange = moderate, yellow = least significant. Red stars indicate the special interest Go terms. (**D**) Heat map showing the expression of up-regulated genes, which contribute to enrichment of these special interest GO terms, at the three growth stages. The red-to-blue scale represents a decreasing log_2_-fold change of gene expression in DP compared with SP system.

Go enrichment analysis revealed that ten biological processes were enriched in up-regulated genes in DP leaves at 45 DAF (Fig. 4A and Supplementary Table S3). The up-regulated genes were mainly associated with ‘phenylpropanoid metabolic process’ (GO:0009698), ‘oxidation–reduction process’ (GO:0055114) and ‘carbohydrate derivative catabolic process’ (GO:1901136). Special interest child terms of these GO terms were ‘lignin catabolic process’ (GO:0046274) and ‘glycerol-3-phosphate catabolic process’ (GO:0046168) (Fig. 4A,D). Two *PpG3PDHs* (LOC103927132 and LOC103953210) encoding glycerol-3-phosphate dehydrogenase, two *PpCesAs* (LOC103958831 and LOC103959701) encoding cellulose synthase A and three *PpLACs* (LOC103959581, LOC103949457 and LOC103930925) encoding laccase all contribute to enrichment of these GO terms. Thus, canopy structure of DP appears to establish a microclimate favorable to carbohydrate metabolism, in comparison to SP counterpart. While six GO terms were enriched for down-regulated genes, and they are mostly associated with ‘sulfate reduction’ (Go:0019419) and ‘cell redox homeostasis’ (GO:0045454) (Supplementary Table S3).

Further, we analyzed the enrichment of GO biological terms at 75 DAF stage. The over-represented categories with the highest confidence among the up-regulated genes in the DP leaves were associated with photosynthesis process and nutrient contribution, including ‘photosynthesis, light harvesting’ (Go:0009765), ‘photosynthesis, dark reaction’ (GO:0019685), ‘fatty acid biosynthetic process’ (GO:0006633) and ‘protein transport’ (GO:0015031) (Fig. 4B,D). It is worth noting that as many as 17 genes encoding light-harvesting chlorophyll a/b-binding (LHC) protein were clustered in the DP leaves enriched category ‘photosynthesis, light harvesting’. On the other hand, 65 biological processes were significantly over-represented in the down-regulated DEGs including ‘nucleic acid metabolic process’ (GO:0090304), ‘macromolecule biosynthetic process’ (GO:0009059), ‘phosphorus metabolic process’ (GO:0006793) and ‘serine family amino acid metabolic process’ (GO:0009069) (Supplementary Table S3). This GO enrichment analysis revealed DP canopy microclimate possibly promotes or triggers the light harvesting response and accelerate fatty acid biosynthesis, while the microclimate conditions of SP canopy architecture activate metabolic pathways.

In addition, six and 16 biological processes were highly enriched among the up-regulated and down-regulated DEGs in the DP leaves at 105 DAF, respectively (Fig. 4C and Supplementary Table S3). In the group of up-regulated DEGs, one interesting Go category, ‘pigment metabolic process’ (GO:0042440), was significantly enriched in the DP leaves. Within the down-regulated genes, ‘flavonoid biosynthetic process’ (GO:0009813), ‘nucleic acid metabolic process’ (GO:0090304), ‘l-ascorbic acid biosynthetic process’ (GO:0019853) and ‘brassinosteroid homeostasis’ (GO:0010268), were significantly enriched categories in the SP leaves.

To identify biological pathways regulated by the distinct training systems, the KEGG enrichment analysis was performed. Our results showed that a total of six and nine pathways were significant enriched in DP leaves at 45 DAF and 75 DAF stages, respectively, but no up-regulated genes were significantly enriched in any pathway at 105 DAF stage (Supplementary Table S4). These enriched pathways included ‘amino sugar and nucleotide sugar metabolism’ (ko00520), ‘photosynthesis’ (ko00195), ‘photosynthesis-antenna proteins’ (ko00196), ‘carbon fixation in photosynthetic organisms’ (ko00710) and ‘fatty acid elongation’ (ko00062). In the other hand, the down-regulated genes were grouped into two, two and five significantly enriched pathways at the three stages, respectively. These pathways included ‘circadian rhythm-plant’ (ko04712) and ‘Plant–pathogen interaction’ (ko04626) in our study.

To investigate potential regulators of transcription factors (TFs) mediating light signaling, up-regulated and down-regulated genes were examined for the over-representation of TFs families, respectively. In total, 89 putative TFs which were up-regulated in DP system at least one developmental stage were identified and grouped into 24 TFs families (Supplementary Figure S2). AP2/ERF TFs constituted the largest group (11 genes, 12.4%), followed by bHLH (10 genes, 11.2%), MYB_related (9 genes, 10.1%), C2C2 (6 genes, 6.7%) and SBP (6 genes, 6.7%). A total of 153 TFs were significantly down-regulated and classified into 20 putative TF families. Among them, WRKY represented the most abundant category (29 genes, 19.0%), followed by AP2/ERF (27 genes, 17.6%), NAC (22 genes, 14.4%), MYB (15 genes, 9.8%), GRAS (10 genes, 6.5%) and HSF (10 genes, 6.5%).

### WGCNA analysis reveals potential genes associated with photosynthetic performance under distinct training systems

**Figure 5:**
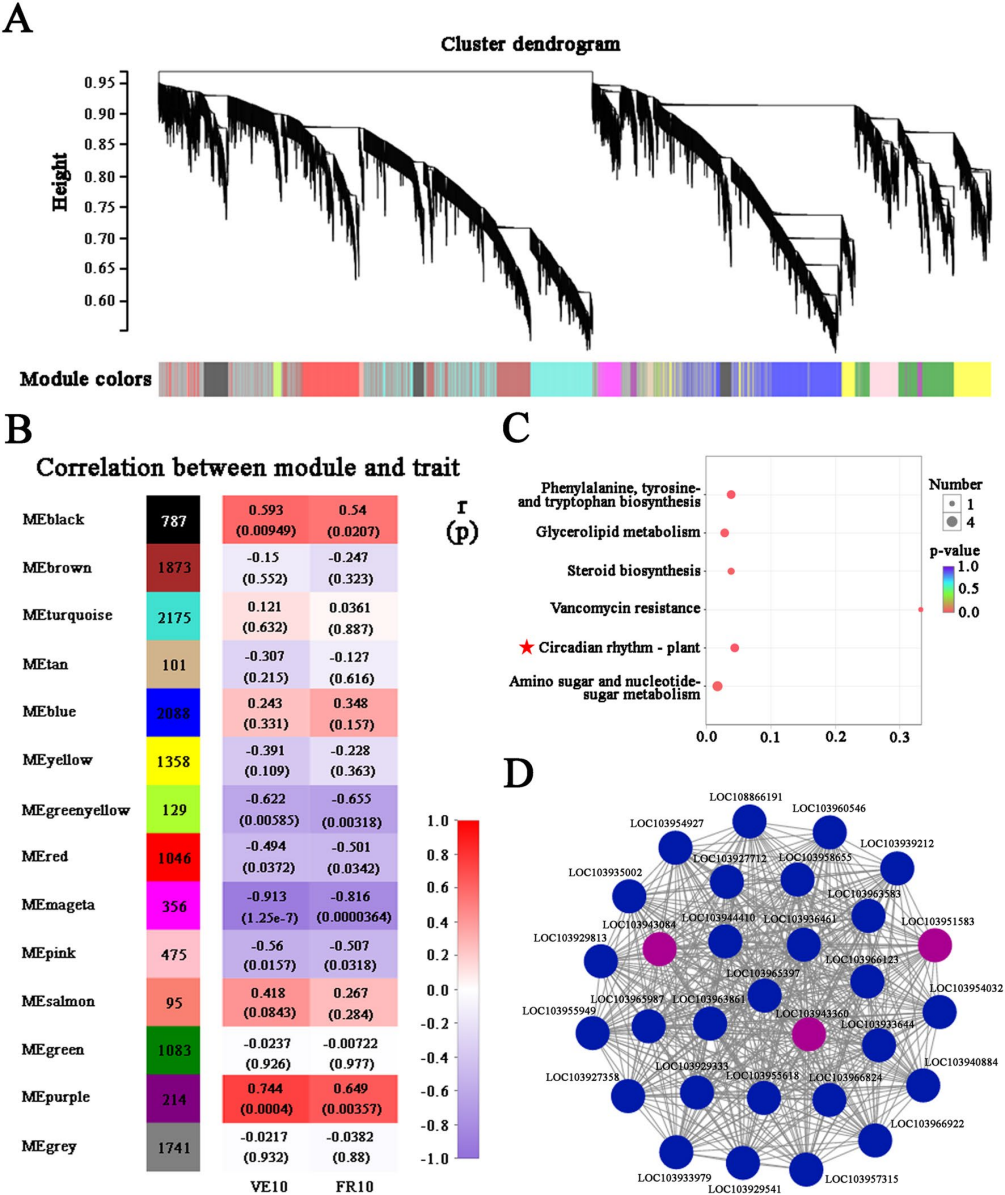
Weighted gene co-expression network analysis (WGCNA) of all expressed genes in pear. (**A**) Hierarchical clustering tree showing co-expression modules identified by WGCNA. Genes are represented by vertical lines (leaves) on the X-axis grouped into branches. Network distance is given on the Y-axis. Cluster color below denotes the separation of leaves into discrete modules. (**B**) Correlation heat map of module-trait association. The left lane indicates 14 modules along with the number of gene belonging to each module. Each cell of right lane contains the correlation coefficient (r) and the *p* value of the module-trait association. The red-to-purple scale indicates decreasing correlation levels, with red representing positive correlation and purple representing negative correlation. (**C**) Bubble plot of enriched KEGG pathways for the purple module (*p* value < 0.05). Bubble color and size correspond to the *p* value and gene number enriched in the pathway. The rich factor indicates the ratio of the number of genes in purple module mapped to a certain pathway to the background number of genes mapped to this pathway. (**D**) Connectivity map of the top 30 connected genes in the purple module. Each node represents a hub gene. Purple nodes represent genes involved in ‘Circadian rhythm-plant’ pathway.

To further investigate candidate genes related to photosynthetic performance under distinct training systems, we performed a WGCNA analysis with all expression genes. A total of 14 distinct modules with sizes ranging from 95 to 2175 genes were identified (Fig. 5A,B). Analysis of the module-trait relationships revealed that the purple module comprising 214 genes showed strong positive correlation with *P*_N_ value of fruiting leaves (r = 0.744, *p* = 0.0004) and vegetative counterparts (r = 0.649, *p* = 0.00357), indicating that expression of genes in this module may be related to photosynthetic performance. No significantly enriched KEGG pathway in purple module can be observed but six pathways showed near-enriched (*p* value < 0.05) (Fig. 5C). For better visualization of the intramodular connectivity, the top 30 network hub genes of the highest network degree in the purple module were identified (Fig. 5D). Interestingly, of these network hub genes, *PpFKF1* (LOC103943084, interconnectivity degree = 51.70) and *PpPRR5* (*Pseudo Response Regulator 5*; LOC103943360, interconnectivity degree = 48.73) were the most interconnected genes. We noted that the other *PpPRR5* (LOC103951583, interconnectivity degree = 42.40) also had high interconnectivity. All these three DEGs involved in the ‘Circadian rhythm-plant’ pathway (KEGG pathway id: ko04712; *p* value = 0.006, FDR = 0.230), implying that circadian clock may play key roles in photosynthetic performance under distinct canopy microclimate.

### Effects of canopy conditions on photosynthetic pigments, PNUE, soluble protein and PpRCA enzyme activity

**Figure 6:**
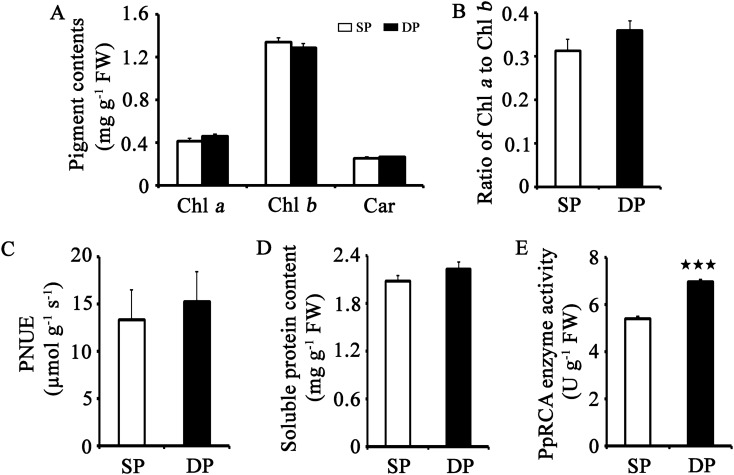
Contents of photosynthetic pigments, soluble protein, Rubisco activase (RCA) and photosynthetic nitrogen-use efficiency (PNUE) between distinct training systems. (**A**) Contents of chlorophyII (Chl) *a*, Chl *b*, carotenoids (Car), and Chl *a* to Chl *b* ratio (Chl *a*/*b*). (**B**) Soluble protein content. (**C**) RCA content. (**D**) PNUE content. Bars indicate standard deviation (n = 8). Data are presented as mean ± stand error of the mean. Asterisks indicated significance as follows: ★★★*p* ≤ 0.001, ★★0.001 < *p* ≤ 0.01 and ★0.01 < *p* ≤ 0.05 as determined using Student’s *t*-test.

GO analysis indicated that the canopy structure of flat-type trellis system represents an effective strategy for increasing expression of genes that potentially contribute to ‘photosynthesis, light harvesting’ (Go:0009765), ‘photosynthesis, dark reaction’ (GO:0019685), ‘carbohydrate derivative catabolic process’ (GO:1901136), ‘protein localization’ (GO:0008104) and ‘pigment metabolic process’ (GO:0042440) (Fig. 4). To examine the effects of canopy conditions on photosynthetic physiology and dry matter accumulation in the field canopies, we investigated photosynthetic pigments, PNUE levels, soluble protein, and PpRCA enzyme activity between distinct training systems (Fig. 6). Compared to SP system, the activity level of PpRCA enzyme was significantly higher in the leaves of DP system. Although no significant differences were observed, the contents of Chl *a* and carotenoids, PNUE value, soluble protein content, the ratio of Chl *a* to Chl *b* (Chl *a*/*b*) tended to be higher in DP system, while the content of Chl *b* showed an opposite trend.

## Discussion

Canopy architecture is critical in determining the light distribution, and therefore the photosynthetic productivity of crop. However, information about the mechanism of impact of training systems on photosynthesis is limited. In this study, both GO and KEGG analysis indicated that the up-regulated genes in DP training system participate in photosynthesis processes such as light harvesting (Fig. 4, Supplementary Table S3 and Table S4). The over-representation of genes in these categories was an expected result because of the higher photosynthetic levels in DP systems (Fig. 2). Light harvesting is the first step in the process of photosynthesis, which is regulated by physiological status and environmental signals^43^. LHC proteins principal roles are to efficiently collect light energy and provide photoprotection, which associated with both short- and long-term adaptations to changing environments, such as fluctuating light intensity, temperature changes, or nutrient availability^43–46^. Some studies indicated that expression levels of *LHC* genes were induced in response to high light, suggesting a photoprotective function for these genes products^47,48^. Compared with traditional freestanding system with relatively shaded conditions, the flat-type trellis system allows trees to receive sunlight uniformly and opulently^9^. In the natural environment, DP leaves may suffer excess light that may damage their photosystems, especially in summer (75 DAF-105 DAF). In the present study, 17 *PpLhc* mRNAs accumulated in the DP leaves with abundant light conditions (Fig. 4D), suggesting a more significant photoprotection and/or light capture roles for DP leaves by enhancing its light-harvesting potential. In higher plants, most photosynthetic pigments (Chls and carotenoids) are coordinated by LHC proteins^49^. Rapid qualitative adjustment in leaf photosynthetic apparatus and photosynthetic capacity can occur in response to short alterations in light availability^50^. As a major change, sun-exposed plants tends to increase the Chl *a*/*b* ratio^51^. This general finding was also confirmed in the current study, as a higher photosynthetic levels and Chl *a*/*b* ratio were observed in DP leaves with relatively higher irradiance (Figs. 2 and 6B and Supplementary Figure S1B). All these data support that canopy structure of DP is well proportioned and maximize light capture to improve photosynthetic efficiency, which may accelerate nutrient accumulation and transport.

Improving the performance of the carbon fixation has the potential to significantly enhance photosynthetic efficiency and crop productivity^52,53^. It is documented that increasing PNUE of the leaves may improve dry matter accumulation^54^. Our results showed that DP system had higher PNUE values and soluble protein contents (Fig. 6C,D), indicating that carbon fixation process may be activated in DP system. Rubisco, which is composed if the eight large subunits (RbcLs) and eight small subunits (RbcSs), is the key enzyme catalyzing the first step of photosynthetic carbon assimilation through the dark reaction^55,56^. Of these, RbcS is thought to be indirectly involved in the catalytic reaction^56^. Overexpression of *RbcS* genes showed to enhance the catalytic performance of Rubisco, suggesting that *RbcS* could be an important factor in determining kinetic properties of Rubisco^55,57^. In this study, the GO term ‘photosynthesis, dark reaction’ was also enriched in DP leaves (Fig. 4B). Three *PpRbcS* genes (LOC103948502, LOC103948503 and LOC1039346 23) were found to be up-regulated in the DP leaves compared to the SP leaves (Figs. 3E and 4D), which may greatly enhance the catalytic performance of Rubisco and contribute to increase dark reaction activity. We also provide evidence that the activate level of PpRCA enzyme was significantly higher in DP system (Fig. 6E). It is long known that the activation state of Rubisco is regulated by RCA via the maintenance of Rubisco catalytic sites^58,59^. Consequently, PpRCA activity might play an important role in the regulation of *PpRbcS* genes and photosynthetic carbon assimilation.

In DP system, we noticed a significantly increased expression of genes that were involved in primary metabolism such as carbohydrate derivative catabololic process (GO:1901136), fatty acid and monocarboxylic acid biosynthesis (GO: 0006633 and 0072330) and protein transport (GO:0015031) (Fig. 4), which indicated a more adequate carbohydrate accumulation and utilization for DP system. Though both the leaves in the interior part of the canopy were investigated in the two training system, DP leaves seem to expose to much more irradiance. As compared to shady leaves, sun leaves are often characterized by a higher cutin, lipid and starch content per dry weight basis, and soluble carbohydrate levels^60^. In this study, *PpKCS* genes encoding 3-ketoacyl-CoA synthase (LOC103947136, LOC103949661, LOC103966995, LOC103928579, LOC108865655, LOC103948450 and LOC103947523) which catalyzes the first reaction in fatty acid elongation and plays a role in cutin and wax biosynthesis^61,62^, were higher expressed in DP leaves (Fig. 4D). Lipids are the major constituents of all membranous structures in plants^63^. The abundant thylakoid membranes in the chloroplast are the site of the photosynthesis light reactions^64^. Lipid biosynthesis genes *PpG3PDH* (LOC103953210 and LOC103927132) which catalyzes the formation of the backbone of membrane lipids also exhibited higher expression levels in DP system leaves (Figs. 3E and 4D). Overexpression of *AtG3PDH* gene showed significant enhancement of plastidic lipid content and photosynthetic efficiency^63^. These results imply that enhancement of PpKCS and PpG3PDH activity in DP leaves may promote to lipid accumulation that greatly influences photosynthetic efficiency.

Certain stress-assoiated transcription factor families, such as WRKY and NAC genes, have been demonstrated to participate in variable light conditions^65,66^. For example, the *AtWRKY22* was found to participate in the dark-induced senescence signal pathway^67^. The ectopic overexpression of *TaWRKY7* in Arabidopsis significantly promoted early leaf senescence under darkness treatment^68^. Whole-genome ATH1 Genome Array studies showed that more than one quarter of NAC proteins in Arabidopsis leaves was up-regulated under dark treatment^69^. In accordance to with these observations, increased expression levels of 29 *PpWRKY* and 22 *PpNAC* TFs were showed in SP system with lower light intensity compared with the DP system (Supplementary Figure S2). These results implied that senescence-associated TFs, the WRKY and NAC superfamilies, may play crucial roles in regulating, acclimating, and modulating gene expression in photosynthesis process in response to low light. In the other hand, when shaded by neighbouring vegetation, plants are exposed to a variety of light quality signal^70^. *PIF* TFs have been demonstrated as positive regulators of shade avoidance^71^. The expression levels of two PIF TFs, *PpPIF4* (LOC103947396) and *PpPIF7* (LOC103931008), were significantly induced in DP system with open-canopy characteristics (Fig. 3E and Supplementary Figure S2). It may suggest that a possible feedback mechanisms mediated by complicated light signaling network may exist due to highly dynamic canopy microclimate.

A new insight into the application of WGCNA and the enrichment of the key module led to the identification of a central role for circadian rhythm regulation related to photosynthetic performance under distinct training system (Fig. 5). Light availability varies not only across the course of the day but also with the changes of canopy architecture, which may provide complex signals to the circadian clock^72^. Recent studies have discovered a link between altered circadian clock regulation and increased levels of photosynthetic activities and biomass in plants^73–75^. For example, maize circadian clock genes *ZmCCA1s* are diurnally up-regulated in the hybrids and target thousands of output genes early in the morning-phased genes; consequently the bindings to carbon fixation genes promote photosynthesis and growth vigor^75^. In our study, network construction highlighted hub genes *PpPRR5* and *PpFKF1*, which showed positive correlation with photosynthetic performance under distinct training systems, predicted to play important roles in circadian rhythm (Fig. 5C,D). *Arabidopsis* homologue *AtPRR5*, together with other core circadian clock components, form complicated transcription feedback loops that mediating a number of circadian outputs^76,77^. “Red or far-red light signaling pathway” was reported to be an enriched category in *AtPRR5* direct-targets^78^. AtFKF1 is a blue light photoreceptor and could target AtPRR5 for degradation^79^. In nature, different canopy conditions combine changes in light spectral quality^50^. The red: far red and blue levels of daylight decreases as it passes through the vegetative canopy^80,81^. In our study, the expression levels of *PpPRR5* and *PpFKF1* were up-regulated in SP system with mutual shading characteristics. It would be reasonable to speculate that the varied light signal are pronounced at highly dynamic and heterogeneous light conditions, and thus energetically activate the temporal regulation of circadian clock. The oscillator components of the clock, *PpPRR5* and *PpFKF1*, may modulate pear leaves response to light cues and cause a cascade of effects on downstream regulatory pathways, leading to photosynthetic variability.

## Supplementary information

Supplementary Information.

Supplementary Table S1.

Supplementary Table S2.

Supplementary Table S3.

Supplementary Table S4.

## Data Availability

All materials and data sets represented in the current study are available in the main text or the supplementary materials. RNA-Seq data are deposited in the Sequence Read Archive (SRA) database (the accession number PRJNA579772) at the National Centre for Biotechnology Information (NCBI). The metadata are available at https://www.ncbi.nlm.nih.gov/bioproject/PRJNA579772.

## References

[1] Cejudo FJ, Ojeda V, Delgado-Requerey V, González M, Pérez-Ruiz JM (2019). Chloroplast redox regulatory mechanisms in plant adaptation to light and darkness. Front. Plant Sci..

[2] Martel AB, Qaderi MM (2017). Light quality and quantity regulate aerobic methane emissions from plants. Physiol. Plant.

[3] Feng L (2019). The influence of light intensity and leaf movement on photosynthesis characteristics and carbon balance of soybean. Front. Plant Sci..

[4] Szymańska R, Ślesak I, Orzechowska A, Kruk J (2017). Physiological and biochemical responses to high light and temperature stress in plants. Environ. Exp. Bot..

[5] Natali A, Croce R (2015). Characterization of the major light-harvesting complexes (LHCBM) of the green alga *Chlamydomonas reinhardtii*. PLoS ONE.

[6] Okello RCO, de Visser PHB, Heuvelink E, Marcelis LFM, Struik PC (2016). Light mediated regulation of cell division, endoreduplication and cell expansion. Environ. Exp. Bot..

[7] Ishii H, Asano S (2010). The role of crown architecture, leaf phenology and photosynthetic activity in promoting complementary use of light among coexisting species in temperate forests. Ecol. Res..

[8] Jaio A (2014). Impact of tree training system, branch type and position in the canopy on the ripening homogeneity of ‘Abbé Fétel’ pear fruit. Tree Genet. Genom..

[9] Liu Z (2018). Selection and validation of suitable reference genes for qRT-PCR analysis in pear leaf tissues under distinct training systems. PLoS ONE.

[10] Tang L (2015). Light interception efficiency analysis based on three-dimensional peach canopy models. Ecol. Inform..

[11] Araujo WL (2008). Limitations to photosynthesis in coffee leaves from different canopy positions. Plant Physiol. Biochem..

[12] Zhang J, Serra S, Leisso RS, Musacchi S (2016). Effect of light microclimate on the quality of ‘d’Anjou’ pears in mature open-centre tree architecture. Biosyst. Eng..

[13] Yao H (2015). Plant density alters nitrogen partitioning among photosyntheticcomponents, leaf photosynthetic capacity and photosynthetic nitrogen use efficiency in field-grown cotton. Field Crops Res..

[14] Gu J (2017). Canopy light and nitrogen distributions are related to grain yield andnitrogen use efficiency in rice. Field Crops Res..

[15] Nebauer SG, Renau-Morata B, Guardiola JL, Molina R (2011). Photosynthesis down-regulation precedes carbohydrate accumulation under sink limitation in *Citrus*. Tree Physiol..

[16] Quentin AG, Close DC, Hennen LMHP, Pinkard EA (2013). Down-regulation of photosynthesis following girdling, but contrasting effects on fruit set and retention, in two sweet cherry cultivars. Plant Physiol. Biochem..

[17] Proietti P (2000). Influence of leaf position, fruit and light availability on photosynthesis of two chestnut genotypes. Sci. Hortic..

[18] Duan W (2016). Genome-wide transcriptional profile analysis of *Prunus persica* in response to low sink demand after fruit removal. Front. Plant Sci..

[19] Ding Z (2016). Transcriptome response of cassava leaves under natural shade. Sci. Rep..

[20] Ruban AV (2015). Evolution under the sun: optimizing light harvesting in photosynthesis. J. Exp. Bot..

[21] Mathur S, Jain L, Jajoo A (2018). Photosynthetic efficiency in sun and shade plants. Photosynthetica.

[22] Shibata K, Koizumi K, Seki T, Kitao I, Matsushita K (2008). A “joint tree” training system enables early returns on Japanese pear orchards. Acta Hortic..

[23] Goyal A (2016). Shade promotes phototropism through phytochrome B-controlled auxin production. Curr. Biol.

[24] Wang Y (2018). Transcriptomic analysis of field-grown rice (*Oryza sativa* L.) reveals responses to shade stress in reproductive stage. Plant Growth Regul..

[25] Klem K (2019). Distinct morphological, physiological, and biochemical responses to light quality in barley leaves and roots. Front. Plant Sci..

[26] Hu B, Liu H, Wang Y (2016). Investigation of the variability of photosynthetically active radiation in the Tibetan Plateau, China. Renew. Sust. Energ. Rev..

[27] Park Y, Runkle ES (2018). Far-red radiation and photosynthetic photon flux density independently regulate seedling growth but interactively regulate flowering. Environ. Exp. Bot..

[28] Oakenfull RJ, Davis SJ (2017). Shining a light on the *Arabidopsis circadian* clock. Plant Cell Environ..

[29] Ni M, Tepperman JM, Quail PH (1998). PIF3, a Phytochrome-interacting factor necessary for normal photoinduced signal transduction, is a novel basic helix–loop–helix protein. Cell.

[30] Martínez-García JF, Huq E, Quail PH (2000). Direct targeting of light signals to a promoter element-bound transcription factor. Science.

[31] Feng D, Wang Y, Lu T, Zhang Z, Han X (2017). Proteomics analysis reveals a dynamic diurnal pattern of photosynthesis-related pathways in maize leaves. PLoS ONE.

[32] Liu Z (2018). Overexpression of the *CsFUS3* gene encoding a B3 transcription factor promotes somatic embryogenesis in *Citrus*. Plant Sci..

[33] Wu J (2013). The genome of the pear (*Pyrus bretschneideri* Rehd.). Genome Res..

[34] Mortazavi A, Williams BA, McCue K, Schaeffer L, Wold B (2008). Mapping and quantifying mammalian transcriptomes by RNA-Seq. Nat. Methods.

[35] Love MI, Huber W, Anders S (2014). Moderated estimation of fold change and dispersion for RNA-seq data with DESeq2. Genome Biol..

[36] Chen H, Boutros PC (2011). VennDiagram: a package for the generation of highly-customizable Venn and Euler diagrams in R. BMC Bioinform..

[37] Klopfenstein DV (2018). GOATOOLS: a python library for gene ontology analyses. Sci. Rep..

[38] Kanehisa M, Goto S (2000). KEGG: kyoto encyclopedia of genes and genomes. Nucleic Acids Res..

[39] Kanehisa M, Sato Y, Furumichi M, Morishima K, Tanabe M (2019). New approach for understanding genome variations in KEGG. Nucleic Acids Res..

[40] Kanehisa M (2019). Toward understanding the origin and evolution of cellular organisms. Protein Sci..

[41] Langfelder P, Horvath S (2008). WGCNA: an R package for weighted correlation network analysis. BMC Bioinform..

[42] Jiang C, Zu C, Wang H (2015). Effect of nitrogen fertilization on growth and photosynthetic nitrogen use efficiency in tobacco (*Nicotiana tabacum* L.). J. Life Sci..

[43] Silva J (2016). Molecular characterization of 5-chlorophyll a/b-binding protein genes from *Panax ginseng* Meyer and their expression analysis during abiotic stresses. Photosynthetica.

[44] Grewe S (2014). Light-harvesting complex protein LHCBM9 is critical for photosystem II activity and hydrogen production in *Chlamydomonas reinhardtii*. Plant Cell.

[45] Rochaix JD (2014). Regulation and dynamics of the light-harvesting system. Annu. Rev. Plant Biol..

[46] Zou Z, Yang J (2019). Genomics analysis of the light-harvesting chlorophyll a/b-binding (Lhc) superfamily in cassava (*Manihot esculenta* Crantz). Gene.

[47] Floris M, Bassi R, Robaglia C, Alboresi A, Lanet E (2013). Post-transcriptional control of light-harvesting genes expression under light stress. Plant Mol. Biol..

[48] Guan Z (2016). Identification and expression analysis of four light harvesting-like (*Lhc*) genes associated with light and desiccation stress in *Ulva linza*. J. Exp. Mar. Biol. Ecol..

[49] Wientjes E, Roest G, Croce R (2012). From red to blue to far-red in Lhca4: How does the protein modulate the spectral properties of the pigments?. Biochim. Biophys. Acta.

[50] Hallik L, Niinemets U, Kull O (2012). Photosynthetic acclimation to light in woody and herbaceous species: a comparison of leaf structure, pigment content and chlorophyll fluorescence characteristics measured in the field. Plant Biol..

[51] Kornarzyński K, Dziwulska-Hunek A, Kornarzyńska-Gregorowicz A, Sujak A (2018). Effect of electromagnetic stimulation of amaranth seeds of different initial moisture on the germination parameters and photosynthetic pigments content. Sci. Rep..

[52] Wilson RH, Alonso H, Whitney SM (2016). Evolving *Methanococcoides burtonii* archaeal Rubisco for improved photosynthesis and plant growth. Sci. Rep..

[53] Bar-Even A (2018). Daring metabolic designs for enhanced plant carbon fixation. Plant Sci..

[54] Chen YL (2014). Characterization of the plant traits contributed to high grain yield and high grain nitrogen concentration in maize. Field Crops Res..

[55] Morita K, Hatanaka T, Misoo S, Fukayama H (2014). Unusual small subunit that is not expressed in photosynthetic cells alters the catalytic properties of Rubisco in rice. Plant Physiol..

[56] Pottier M, Gilis D, Boutry M (2018). The hidden face of Rubisco. Trends Plant Sci..

[57] Ishikawa C, Hatanaka T, Misoo S, Miyake C, Fukayama H (2011). Functional incorporation of sorghum small subunit increases the catalytic turnover rate of Rubisco in transgenic rice. Plant Physiol..

[58] Wei L, Wang Q, Xin Y, Lu Y, Xu J (2017). Enhancing photosynthetic biomass productivity of industrial oleaginous microalgae by overexpression of RuBisCO activase. Algal Res..

[59] Zhao G, Xu H, Zhang P, Su X, Zhao H (2017). Effects of 2,4-epibrassinolide on photosynthesis and Rubisco activase gene expression in *Triticum aestivum* L. seedlings under a combination of drought and heat stress. Plant Growth Regul..

[60] Lichtenthaler HK (1981). Photosynthetic activity, chloroplast ultrastructure, and leaf characteristics of high-light and low-light plants and of sun and shade leaves. Photosynth. Res..

[61] Wang X (2017). A β-ketoacyl-CoA synthase is involved in rice leaf cuticular wax synthesis and requires a CER2-LIKE protein as a cofactor. Plant Physiol..

[62] Su W, Ye C, Zhang Y, Hao S, Li QQ (2019). Identification of putative key genes for coastal environments and cold adaptation in mangrove *Kandelia obovata* through transcriptome analysis. Sci. Total Environ..

[63] Singh V (2016). Over-expression of *Arabidopsis thaliana SFD1/GLY1*, the gene encoding plastid localized glycerol-3-phosphate dehydrogenase, increases plastidic lipid content in transgenic rice plants. J. Plant. Res..

[64] Schneider A (2016). The evolutionarily conserved protein photosynthesis affected MUTANT71 is required for efficient manganese uptake at the thylakoid membrane in *Arabidopsis*. Plant Cell.

[65] Chen L (2012). The role of WRKY transcription factors in plant abiotic stresses. Biochim. Biophys. Acta..

[66] Hu R (2010). Comprehensive analysis of NAC domain transcription factor gene family in *Populus trichocarpa*. BMC Plant Biol..

[67] Zhou X, Jiang J, Yu D (2011). WRKY22 transcription factor mediates dark-induced leaf senescence in *Arabidopsis*. Mol. Cells.

[68] Zhang H (2016). Identification and function analyses of senescence-associated WRKYs in wheat. Biochem. Biophys. Res. Commun..

[69] Lin J, Wu S (2004). Molecular events in senescing *Arabidopsis* leaves. Plant J..

[70] Franklin KA (2008). Shade avoidance. New Phytol..

[71] Leivar P, Quail PH (2011). PIFs: pivotal components in a cellular signaling hub. Trends Plant Sci..

[72] Shin J, Anwer MU, Davis SJ (2013). Phytochrome-interacting Factors (PIFs) as bridges between environmental signals and the circadian clock: diurnal regulation of growth and development. Mol. Plant.

[73] Ni Z (2009). Altered circadian rhythms regulate growth vigour in hybrids and allopolyploids. Nature.

[74] Ng DW (2014). A role for CHH methylation in the parent-of-origin effect on altered circadian rhythms and biomass heterosis in *Arabidopsis* intraspecific hybrids. Plant Cell.

[75] Ko DK (2016). Temporal shift of circadian-mediated gene expression and carbon fixation contributes to biomass heterosis in maize hybrid. PLoS Genet..

[76] Resco de Dios V, Gessler A (2018). Circadian regulation of photosynthesis and transpiration from genes to ecosystems. Environ. Exp. Bot..

[77] Li B (2019). PRR5, 7 and 9 positively modulate TOR signaling-mediated root cell proliferation by repressing *TANDEM ZINC FINGER 1* in *Arabidopsis*. Nucleic Acids Res..

[78] Nakamichi N (2012). Transcriptional repressor PRR5 directly regulates clock-output pathways. Proc. Natl. Acad. Sci. U.S.A..

[79] Baudry A (2010). F-Box proteins FKF1 and LKP2 act in concert with ZEITLUPE to control *Arabidopsis* clock progression. Plant Cell.

[80] Yang F (2018). Effect of interactions between light intensity and red-to- far-red ratio on the photosynthesis of soybean leaves under shade condition. Environ. Exp. Bot..

[81] Boccaccini A (2020). Low blue light enhances phototropism by releasing cryptochrome1-mediated inhibition of *PIF4* expression. Plant Physiol..

