# Supplementary material for: Comparative physiological and transcriptomic analysis of pear leaves under distinct training systems
Source: Sci Rep. 2020 Nov 3;10:18892. doi: 10.1038/s41598-020-75794-z (PMC7641215; doi:10.1038/s41598-020-75794-z)
Supplement: Supplementary file 1 — Supplementary Information. [file 41598_2020_75794_MOESM1_ESM.docx]

**Comparative physiological and transcriptomic analysis of pear leaves under distinct training systems**

Zheng Liu^1^*, Liyuan An^1,2^, Shihua Lin^1,2^, Tao Wu^1^, Xianming Li^1^, Junfan Tu^1^, Fuchen Yang^1^, Hongyan Zhu^1^, Li Yang^1^, Yinsheng Cheng^1^, Zhongqi Qin^1,2^*

1 Research Institute of Fruit and Tea, Hubei Academy of Agricultural Sciences, Wuhan 430064, China

2 College of Life Sciences, Wuhan University, Wuhan 430072, China

Author for correspondence

Email: [liuzhenghzau@aliyun.com](mailto:liuzhenghzau@aliyun.com) (Zheng Liu); [zhongqiqin@163.com](mailto:zhongqiqin@163.com) (Zhongqi Qin)


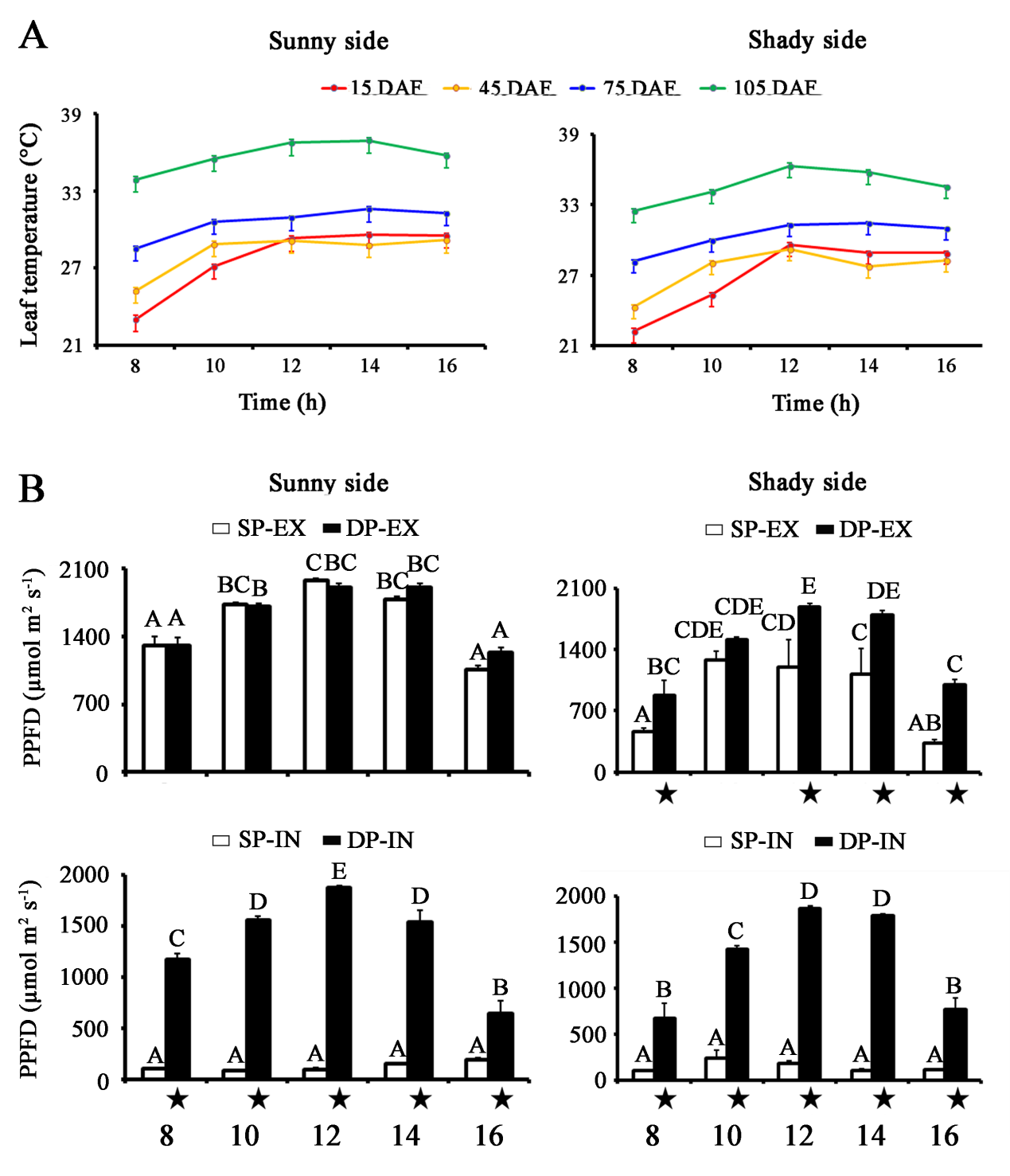


**Supplementary Figure S1.** Diurnal variations in leaf temperature and photosynthetic photon flux density (PPFD). (A) Diurnal change of leaf temperature during four developmental stages. (B) Comparison of diurnal courses of PPFD measured from different canopy locations. SP/DP-EX: the exterior part of the canopy in SP/DP system, SP/DP-IN: the interior part of the canopy in SP/DP system. Each value represents mean ± standard deviation (n = 9). The capital letters above the bars indicted significant differences (P < 0.01). Asterisks below indicate significantly higher levels of PPFD between SP and DP system.


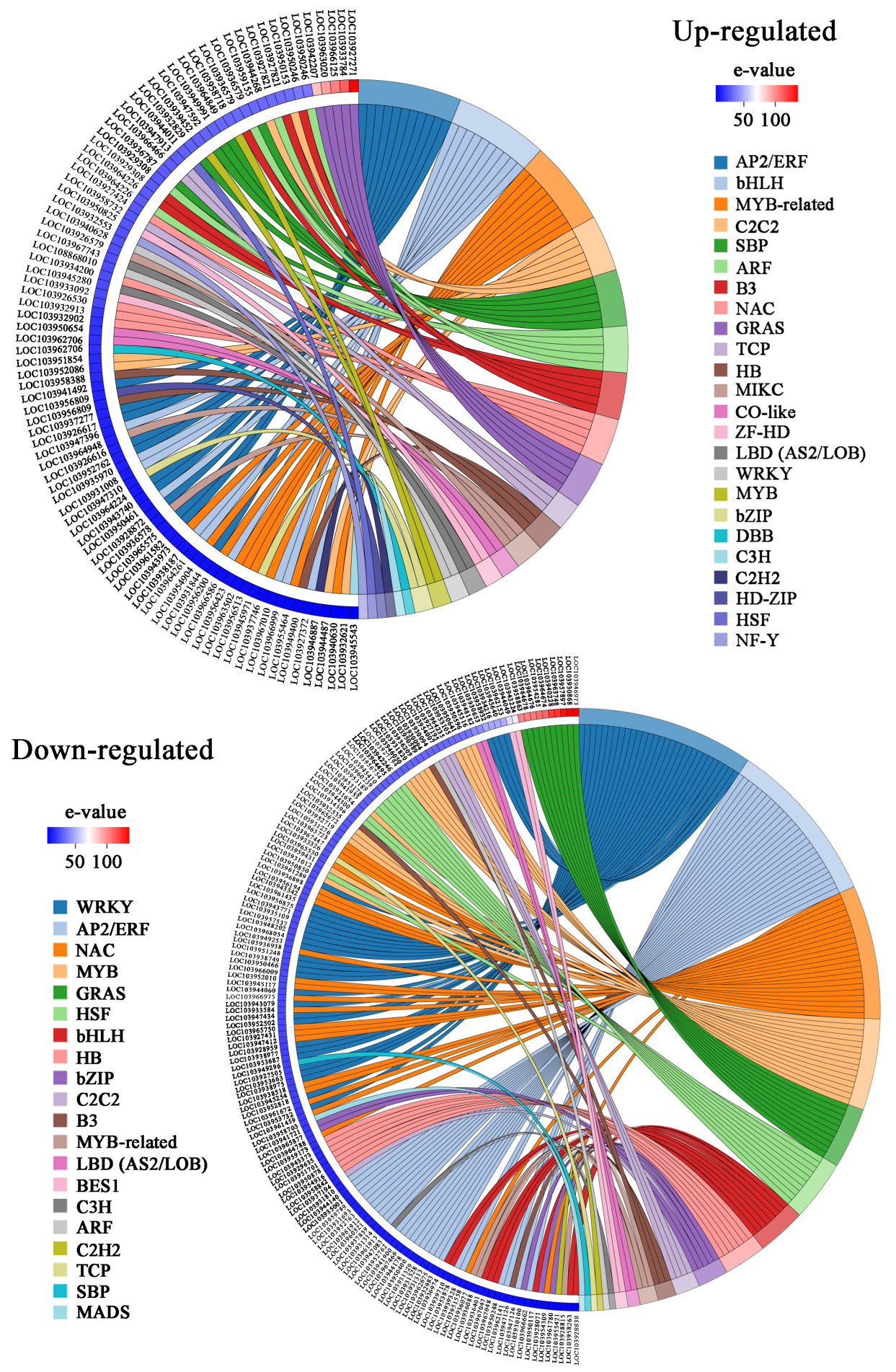


**Supplementary Figure S2**. Chordal graph of differentially expressed transcription factors to families. The blue-to-red scale indicates values change (–log_10_ e-value) from low to high.

# Supplementary Table S1: List of qRT-PCR primers used in this study.

**Supplementary Table S2:** Summary of RNA-seq read statistics.

**Supplementary Table S3:** Gene Ontology (GO) enrichment analysis of differentially expressed genes for biological process.

**Supplementary Table S4:** Statistical enrichment analysis for KEGG pathways (FDR ＜ 0.05).
