# Supplementary material for: Comparative physiological and transcriptomic analysis of pear leaves under distinct training systems
Source: Sci Rep. 2020 Nov 3;10:18892. doi: 10.1038/s41598-020-75794-z (PMC7641215; doi:10.1038/s41598-020-75794-z)
Supplement: Supplementary file 2 — Supplementary Table S1. [file 41598_2020_75794_MOESM2_ESM.doc]

**Supplementary Table S1. List of qRT-PCR primers used in this study.**

| **Gene ID/Name** | **Primer sequences (5’-3’)** | |
| --- | --- | --- |
|  | **Forward** | **Reverse** |
| LOC103927471 | TATTGGAGGATTTGGGTG | AACGGTGATAGGCGAGT |
| LOC103927132 | AGCAGTTGATAGAGCCACA | AGCCTTGCCTCCACATA |
| LOC103953210 | CGGGTCTGTTCACAACT | TCACCATTAGCCTTTCC |
| LOC103948502 | CTCCGCTACCGTGTCTA | CAGTGAATGGAGCAACC |
| LOC103947396 | CTGGCGGTTCTGGTAGT | CAGCAGCCTCACTTTGG |
| LOC103927195 | CTTCTTAGGACTGGCATTG | GGCATATTCTGGGTTGG |
| LOC103931820 | CTGGGTAACAGGTGGTC | TATTTCGTTATCGGTGC |
| LOC103952763 | CGGATTGGGTTGAATGA | GTTGGCGTCGCTGAAGA |
| LOC103960239 | TATTTGCCCGTTGTCTC | ATGCTGGTGTCTTGTATTT |
| *PpSKD1* | CTTCCGCCTCCTATCAC | TTCATCACCCTTCCTCT |
| *PpYLS8* | TGAGGTGCTGGCTTCTGT | TGACCGTTGATGGATCGTA |
